# Urban physical activity for neurogenesis: infrastructure limitations

**DOI:** 10.3389/fpubh.2025.1638934

**Published:** 2025-08-18

**Authors:** Mohamed Hesham Khalil

**Affiliations:** Department of Architecture, University of Cambridge, Cambridge, United Kingdom

**Keywords:** outdoor exercise, urban physical activity, BDNF, hippocampal neurogenesis, brain health, heat stress, environmental health, walking and cycling

## 1 Introduction

Several urban physical activity infrastructure limitations can seriously impair the opportunity of sustaining the daily newborn neurons in the human brain.

Neurogenesis persists in the human brain's hippocampus until the tenth decade of life (1–4). Approximately 700 new neurons are born daily in each hemisphere's hippocampus (5). Nurturing newborn neurons heavily relies on physical activity that increases brain-derived neurotrophic factor (BDNF) (6), which is vital for cognition and mood improvements, and mood regulation (7–10).

Urban infrastructure can increase BDNF through the affordances for physical activity (e.g., walking, cycling) through planning and design (11). However, infrastructure solutions must be aware of the confounding temperature peaks in winter and summer, air pollution, and residential greenness and the tree placement strategy.

## 2 Urban infrastructure affordances for high-intensity physical activity to increase BDNF

The degree to which an environment affords higher metabolic equivalents (>3 METs) increases the likelihood of elevating BDNF in humans (11), where urban environments that support physical activity (e.g., walking and cycling) are more likely to help BDNF in their populations.

For cycling, Gibbons et al. (12) showed through their crossover study that 6 min of vigorous-intensity cycling elevated BDNF in both serum and plasma 4- to 5-fold more than 90 min of light-intensity physical activity. Cycling has been shown to increase serum BDNF and enhance the performance of a face-name-matching task that involves the hippocampus and associated medial temporal lobe structures (13). Similarly, the impact of walking on BDNF has been systematically reviewed (14), where 30 min of walking can be sufficient to increase BDNF (15).

If the environment has walkable pathways and cycling lanes, the affordances for METs increase, and if those lanes have steep slopes, METs can increase from 3 METs up to 16 METs (16), which increases the probability of elevating BDNF to a great extent.

To encourage urban walking and cycling to increase the likelihood of engaging in moderate-to-vigorous physical activity that can increase BDNF. This can be achieved through enhancing the urban infrastructure that encourages physical activity such as through understanding the environmental attributes that promote higher urban physical activity such as residential density, intersection density, public transport density, and number of parks (17), through improving accessibility, connectivity, safety and and the experience of walking and cycling (34).

However, the urban infrastructure affordance for physical activity depends on temperature peaks, pollution, residential greenness, and tree placement.

## 3 Seasonal BDNF differences and urban infrastructure responsivity to geographic temperature peak variances

Urban infrastructure does not face the limitation of affordances for METs alone, but solutions should be based on the geographic dynamics of temperature differences. For instance, Goulet et al. (18) showed that BDNF increased after 180 min of moderate-intensity treadmill walking in a 32°C environment, but not in a 16°C environment. This section discusses how BDNF rises in summer with longer-day periods due to heat increase, but extreme heat waves are likely to cause neuroinflammation that impairs the role of BDNF in nurturing neurogenesis. Those variances urge urban environments to be responsive to their seasonal dynamics.

Summertime is found to be significantly associated with higher BDNF changes. In humans, Molendijk et al. (19) examined seasonal fluctuations in serum concentrations of BDNF among 2,851 participants from the Netherlands Study of Depression and Anxiety (NESDA). The authors demonstrated pronounced elevations of serum BDNF concentrations during the spring and summer months, contrasting markedly with lower concentrations observed during the autumn and winter months. Monthly analyses highlighted substantial variations, with effect sizes (Cohen's d) ranging from moderate to large (0.27–0.66), reinforcing the biological relevance and magnitude of these seasonal differences. Further exploratory analyses illuminated the positive correlations between serum BDNF levels and sunlight exposure. Animal studies support those findings, where Hernandez et al. (20) revealed that hippocampal and hypothalamic BDNF protein peaks in the long-day, euthermic summer and troughs in the short-day, hypothermic winter. The seasonal effects on BDNF, however, could to a great extent be explained by some of the factors discussed subsequently, such as heat stress.

Daytime high temperatures, without severe heat stress, could explain the increase of BDNF in long days during summer. In humans, head-out immersion of males in hot water (42°C) for 20 min increased BDNF (21), and after daylong (9 h) exposure to hot ambient conditions (40°C) in younger and older adults, with slight variation in concentrations between both groups (22). Kirby et al. (23) studied the impact of 22°C (air-conditioned indoor environment), 26°C (recommended indoor temperature limit for health), 31°C, and 36°C (non-air-conditioned home). BDNF was increased by ~ 28% in the latter group compared to the first. They concluded that BDNF increases by 90 pg/mL per 1°C rise in ambient temperature. Animal models support this by explaining how neurogenesis increases in turn. Rats exposed to a 1-h heat treatment (36°C) for 7 days had a 1.4-fold increase in neurogenesis in the hippocampal dentate gyrus compared to controls in a normothermic environment (25°C) (24).

However, severe heat stress induces pathological alterations that encompass oxidative damage and apoptosis of hippocampal neurons and disrupts the BDNF-associated axis (25), which suggests that environments with high temperatures, not leading to severe heat stress, are more likely to increase BDNF.

Hence, the demands from urban infrastructure for physical activity vary accordingly, suggesting that urban planning and design should be geographically sensitive.

## 4 Urbanisation's air pollution inhibits the increase of BDNF via urban physical activity

Though the urban infrastructure can have the appropriate affordance for physical activity (e.g., walking, cycling) that corresponds to the context's temperature dynamics, neurotoxicity caused by air pollution can seriously impair BDNF increase via any form of urban physical activity.

Cycling near a major traffic route did not increase BDNF compared to a similar cycling activity in an air-filtered room due to particulate matter (PM) (26). PM is very common in urban environments (27), affecting brain structure (28–30). On the contrary, indoor physical activity can enhance BDNF levels since it reduces exposure to pollution (35), but household walking is unlikely to reach a moderate-intensity level (16).

Air pollution is a major challenge for most urban environments, making urban physical activity less effective than physical activity in natural environments due to the limited opportunities for walking or cycling, lack of slopes that enhance the effect of physical activity on BDNF, the the predominance of traffic-caused air pollution that is likely to inhibit any potential increase in BDNF via physical activity, which can seriously impair neurogenesis in humans.

## 5 Urban residential greenness, BDNF and tree placement strategies

Greening buildings can counteract the effect of pollution (31), and urban greenness generally overcomes neurotoxicity caused by air pollution as well (32).

Still, greening an urban environment is also geographically-sensitive. There are context-specific greening solutions to harness tree-based cooling in urban environments (33), where the authors found that trees generally cool cities in hot and dry climates, and less in hot and humid climates.

However, in light of the earlier discussion in this paper, reducing peak monthly temperatures to below 26°C can provide a cooling effect through which high-intensity urban physical activity may be needed and not just moderate-intensity walking.

## 6 Conclusion

**Figure 1:**
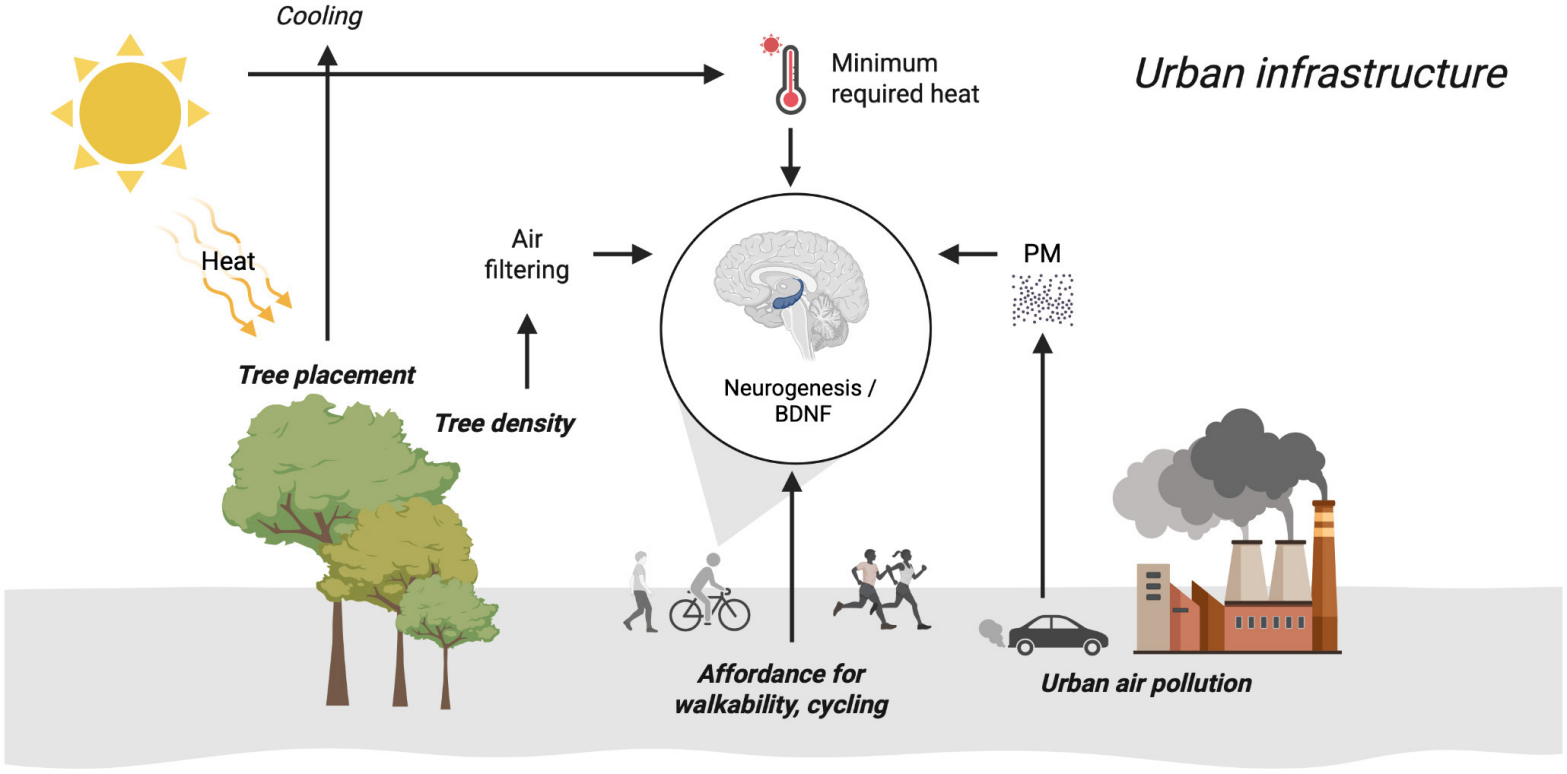
Urban infrastructure for physical activity, neurogenesis, and BDNF.

Supporting neurogenesis in humans via BDNF through urban physical activity is very important. This paper brings the process of urban infrastructure decision making for physical activity to a full circle as illustrated in Figure 1. Several variables determine the effect of urban physical activity on BDNF such as affordance for physical activity, peak temperature is affected by climate change and geographical distributions, impact of temperature variances on the effect of physical activity, air pollution's inhibition of the impact of any potential physical activity even when temperature is taken into consideration, tree cover density counteracting neurotoxicity, and tree placement that can reduce temperatures beyond the effective threshold that increases BDNF.
